# Ripening of Hard Cheese Produced from Milk Concentrated by Reverse Osmosis

**DOI:** 10.3390/foods8050165

**Published:** 2019-05-15

**Authors:** Anastassia Taivosalo, Tiina Kriščiunaite, Irina Stulova, Natalja Part, Julia Rosend, Aavo Sõrmus, Raivo Vilu

**Affiliations:** 1 Center of Food and Fermentation Technologies, Akadeemia tee 15A, 12618 Tallinn, Estonia; tiina@tftak.eu (T.K.); irina.stulova@tftak.eu (I.S.); natalja@tftak.eu (N.P.); julia@tftak.eu (J.R.); aavo@tftak.eu (A.S.); raivo@tftak.eu (R.V.); 2 Department of Chemistry and Biotechnology, Tallinn University of Technology, Akadeemia tee 15, 12618 Tallinn, Estonia

**Keywords:** reverse osmosis, concentrated milk, hard cheese, cheese ripening, volatile compounds

## Abstract

The application of reverse osmosis (RO) for preconcentration of milk (RO-milk) on farms can decrease the overall transportation costs of milk, increase the capacity of cheese production, and may be highly attractive from the cheese manufacturer’s viewpoint. In this study, an attempt was made to produce a hard cheese from RO-milk with a concentration factor of 1.9 (RO-cheese). Proteolysis, volatile profiles, and sensory properties were evaluated throughout six months of RO-cheese ripening. Moderate primary proteolysis took place during RO-cheese ripening: about 70% of α_s1_-casein and 45% of β-casein were hydrolyzed by the end of cheese maturation. The total content of free amino acids (FAA) increased from 4.3 to 149.9 mmol kg^−1^, with Lys, Pro, Glu, Leu, and γ-aminobutyric acid dominating in ripened cheese. In total, 42 volatile compounds were identified at different stages of maturation of RO-cheese; these compounds have previously been found in traditional Gouda-type and hard-type cheeses of prolonged maturation. Fresh RO-cheese was characterized by a milky and buttery flavor, whereas sweetness, saltiness, and umami flavor increased during ripening. Current results prove the feasibility of RO-milk for the production of hard cheese with acceptable sensory characteristics and may encourage further research and implementation of RO technology in cheese manufacture.

## 1. Introduction

The application of a membrane separation technology has gained increasing attention in the dairy industry. The substantial continuing increase in the global milk and cheese production [1] calls for innovations in cheese milk pretreatment to improve the efficiency of cheese manufacturing plants retaining high quality of cheese. Preconcentration of large volumes of raw milk prior to transportation to a cheese plant reduces the delivery costs of milk, and potentially increases the plant capacity and cheese yields.

Ultra- (UF) and microfiltration (MF) for the concentration of cheese milk are the most widely and successfully applied techniques in cheesemaking [2] and have been of enormous research interest. UF retentates have been used to standardize milk protein content by entrapping the whey proteins into the cheese curd matrix to improve the cheese yield and composition of Mozzarella, Cheddar, Camembert, and Brie cheeses [3,4], and semihard cheese made from a mixture of ewes’ and cows’ milk [5]. Fewer studies have been performed on UF-Feta cheese, where the cheese rheological [6] and microstructural changes in fat globules during ripening [7] as well as accelerating the cheese ripening by the addition of lipase have been evaluated [8]. MF retentates with increased casein (CN) content can be added to UF retentates to optimize the cheesemaking process by improving rennet coagulability, which results in a firmer gel and increased cheese yield [2,9]. CN enrichment by MF has been applied to produce Mozzarella [10], Cheddar [11,12], and semihard [13] cheeses with a good sensory quality. The effect of both MF and UF on Edam cheese yield and ripening has been evaluated by Heino et al. [14].

Reverse osmosis (RO) is a filtration method that separates the solutes with a molecular weight of 150 Da and less [2]. Thus, water passes through the membrane, while fats, proteins, lactose, and minerals are retained [2]. The process of RO in the dairy industry has been developed during the last 25 years [15,16] and has received considerable attention, especially that of whey protein concentration [17,18]. Moreover, this dewatering technology has been used for the concentration of skim milk before drying in the production of milk powders [19], and prior to yoghurt manufacture [20]. However, only a limited number of studies have demonstrated the application of RO in cheese technology. Agbevavi et al. [21] made the first attempt to produce Cheddar cheese from whole milk concentrated by RO (RO-milk) on a pilot plant scale. The texture of cheese was assumed to be not uniform because of the high lactose content of the retentate. Moreover, the unacceptable bacterial contamination of the final cheese was observed due to the poor sanitary conditions of the RO system [21]. Barbano and Bynum [22] evaluated the effect of different water reduction levels of whole milk obtained by RO on Cheddar cheese produced in a commercial cheese plant. The authors succeeded in the manufacture of the cheese with increased yield by using conventional cheesemaking equipment. The CN breakdown in Cheddar cheese produced from RO-milk has been shown to be similar to that of the control cheese at earlier stages of ripening, while the slower proteolysis has been noted in aged cheese [23]. Additionally, a significantly higher lactose content in Cheddar cheese produced from RO-milk compared to the cheese from unconcentrated milk was determined, which could cause increased lactic acid fermentation, effecting the sensory properties of cheese. Hydamaka et al. [24] used the whole milk RO retentate to produce the direct acidified cheese with a higher cheese yield and good sensory characteristics. At the moment, there have been no published data on the production and ripening of hard-type cheeses from RO-milk.

In this research, a concentrated milk was manufactured using RO filtration technology. The aim of the study was to produce a hard cheese from the RO-milk (RO-cheese) and to study the impact of milk concentration on cheese ripening. Both mesophilic and thermophilic starter cultures and high curd scalding temperature (48 °C) were used in RO-cheese production. The breakdown of caseins, release of amino acids and volatile compounds, as well as sensory properties of RO-cheese were evaluated during six months of ripening.

## 2. Materials and Methods

### 2.1. RO-Cheese Manufacture and Sampling

A Gouda-type cheese technology with certain modifications into the manufacturing protocol (i.e., addition of thermophilic starter cultures and high scalding temperature) was applied to obtain a cheese with hard texture and low moisture content. A hard cheese was produced in the pilot plant at the School of Service and Rural Economics (Olustvere, Estonia) from 100 L of whole bovine RO-milk (concentration factor of 1.9, dry matter 21.7% w/w, pH 6.44) pasteurized at 74 °C for 15 s. After heat treatment, the RO-milk was cooled to 32 °C and inoculated with mixed starter culture (10 U 100 L^−1^) 30 min prior to addition of microbial rennet (8 g 100 L^−1^; 1300 IMCU g^−1^, Chymax, Chr. Hansen Ltd., Hørsholm, Denmark). The starter consisted of multiple strains of mesophilic and thermophilic lactic acid bacteria (LAB)—FD-DVS CHN-11, FD-DVS LH-B02, and FD-DVS ST-B01 (Chr. Hansen Ltd., Hørsholm, Denmark) mixed in the proportion 10:5:1, respectively—*Lactococcus lactis ssp. lactis*, *Lc. lactis ssp. cremoris*, *Lc. lactis ssp. diacetylactis*, *Leuconostoc sp.*, *Streptococcus thermophilus*, and *Lactobacillus helveticus*. After coagulation, the curd was cut into 0.7 cm × 0.7 cm × 0.7 cm cubes and then slowly stirred for 20 min followed by removing 20 L of whey. Warm water was added to the vat and cheese grains were continuously mixed and heated at 48 °C for 30 min. After pre-pressing at 0.5 bar for 10 min and 1 bar for 10 min in the vat under the whey, the whey was drained off and pre-pressed grain cubes were transferred into low cylinder cheese molds. The cheeses were pressed three times for 20 min at 1, 1.8, and 2.1 bar, and brine-salted (20% NaCl, *w/v*) for 16 h at room temperature. Approximately 1 kg wheel-shaped cheeses were coated with wax (6.6 g kg^−1^ of cheese; Ceska^®^-coat WL01; CSK Food Enrichment, Netherlands) and ripened at 13 °C for six months. Samples were taken from the inner part of the cheese at 0 (fresh cheese before salting), 0.5, 1, 2, 4, and 6 months of ripening and stored at −20 °C until analysis.

### 2.2. Compositional and Microbiological Analyses of Cheeses

Moisture content and pH of the RO-milk and cheeses were measured using a Mettler-Toledo HR83 moisture analyzer (Mettler-Toledo AG, Greifensee, Switzerland) [25] and a pH meter (Mettler-Toledo Ltd., Leicester, UK), respectively. The pH of the cheese was measured by inserting a glass electrode into the compressed grated cheese samples. Total fat content of the cheese was determined by the method of the Association of Official Analytical Chemists AOAC 933.05 [26] in the grated lyophilized cheese samples.

### 2.3. Analysis of Caseins

Fractions of CN and their primary degradation products in cheeses were analyzed by Agilent Capillary Electrophoresis (CE, Agilent Technologies, Waldbronn, Germany) according to the method of Ardö and Polychroniadou [27] as described by Taivosalo et al. [28]. CN fractions were identified and labeled based on the results of Otte et al. [29], Miralles et al. [30], Albillos et al. [31], and Heck et al. [32]. ‘Valley-to-valley’ integration of the peaks was used [30]. The ratio of peak area to the total peak area adjusted to the migration time was used to calculate the relative concentration of caseins in the cheese samples [32].

### 2.4. Analysis of Free Amino Acids

Composition of the free amino acids (FAA) of RO-cheeses was obtained by UPLC (Acquity UPLC, Waters Corp., Milford, MA, USA) controlled by Waters Empower 2.0 software (Waters Corp., Milford, MA, USA) as described by Taivosalo et al. [28]. The absolute concentrations of AA were calculated using standard curves and expressed as mmol kg^−1^ of cheese for total FAA and relative content as mol% for individual FAA.

### 2.5. Analysis of Volatiles by SPME-GC-TOF-MS

The extraction of volatile compounds from RO-cheese samples was carried out using the headspace solid-phase microextraction (HS-SPME) method based on Bezerra et al. [33]. Grated cheese was measured (0.1 g) into a 20 mL glass autosampler vial capped with a PTFE/silicone septum. Vials were preincubated at 40 °C for 5 min. A SPME fiber (30/50 μm DVB/Car/PDMS Stableflex, length 2 cm; Supelco, Bellefonte, PA, USA) was used to extract the volatile compounds from the headspace for 20 min under stirring at 40 °C.

Identification of cheese volatiles was performed using a Micromass GCT Premier gas chromatograph system (Waters, Milford, MA, USA) coupled with a CombiPAL autosampler (CTC Analytics AG, Lake Elmo, MN, USA). After the SPME procedure, the volatile compounds were desorbed in splitless mode into a GC injection port equipped with a 0.75 mm internal diameter liner at 250 °C for 10 min. A DB5-MS column (30 m length × 0.25 mm i.d. × 1.0 μm film thickness; J&W Scientific, Folsom, CA, USA) was used with helium as a carrier gas at a flow rate of 1.0 mL min^−1^. GC conditions were based on the method employed by Lee et al. [34]. The initial oven temperature was 35 °C with a holding time of 3 min. Then, the oven was programmed to ramp-up from 35 °C at a rate of 5 °C min^−1^ to 110 °C, and then from 110 °C at a rate of 10 °C min^−1^ to a final temperature of 240 °C with an additional holding time of 4 min (35 min of total run time). Mass spectra were obtained at ionization energy of 70 eV and a scan speed of 10 scans s^−1^, with a mass-to-charge ratio scan range of 35 to 350. Three analytical replicates were used for each cheese.

Nontargeted identification of volatile compounds was carried out using the ChromaLynx application (version 4.1; MassLynx software; Waters, Milford, MA, USA) and theoretical calculation of Kovats retention indices (RI). Theoretical RI were calculated using the retention times (RT) of the eluting compounds normalized to the RT of adjacent n-alkanes. Accurate identification of the compounds was verified by comparing theoretical RI to the NIST database (US Department of Commerce, Gaithersburg, MD, USA). The quantities of identified compounds were expressed in peak area units (AU) and as a percentage of a peak area against total ion chromatogram (%TIC).

### 2.6. Sensory Analysis

Descriptive sensory analysis was performed by a local sensory panel of eight trained assessors. The testing rooms were in compliance with ISO standard [35]. In total, 35 attributes (11 for odor, five for appearance, 14 for flavor, and five for texture) were assessed on a scale of 0−15. Commercial 6-month-old Old Saare (Saaremaa Dairy Factory, Kuressaare, Estonia), made with both mesophilic and thermophilic starters, and 8-month-old Gouda (Valio Eesti AS, Võru, Estonia) cheeses were chosen as references. A complete list of sensory attributes, attribute definitions, and anchor-values of the reference materials on the scale is presented in Table 1.

For sensory analysis, cheese samples were cut into 6 cm × 1 cm × 1 cm pieces. Three pieces of each sample were served to panel members on a white plate. As an exception, appearance attributes were assessed separately from a cross-section of the cheese wheel. Randomized blind-tasting with sequential monadic order of presentation was used. Two replicate assessments were done for each cheese sample. Water was provided as a palate cleanser between the samples.

## 3. Results and Discussion

A cheese, produced in this study from RO-milk concentrated 1.9-fold on a dry matter basis, was characterized as hard cheese, with the calculated moisture in nonfat substance (MNFS) content of 45.5% (w/w) and fat content of 37.1% (w/w) as determined in 0.5-month-old cheese and pH ranging from 5.10 to 5.23 during ripening for six months.

### 3.1. Proteolysis During RO-Cheese Ripening

**Figure 1:**
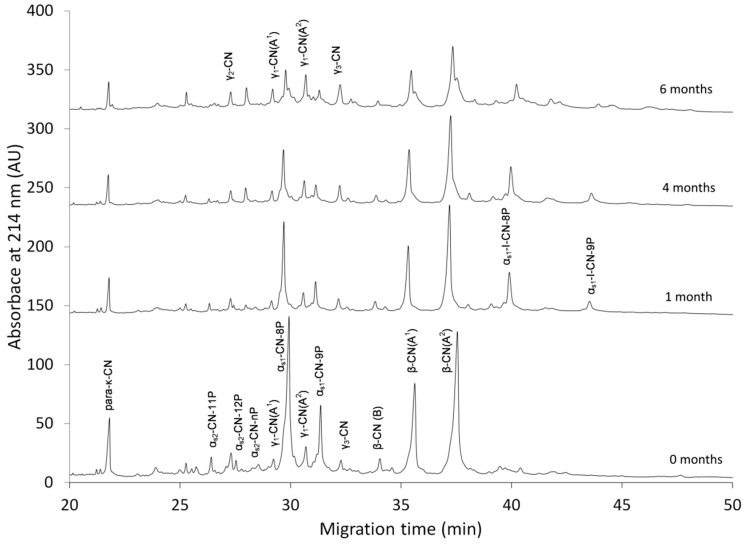
The electropherograms of RO-cheese obtained by CE at 0, 1, 4, and 6 months of ripening. CN: casein; para-κ-CN: κ-CN f1−105; γ_1_-CN: β-CN f29−109; γ_2_-CN: β-CN f106−209; γ_3_-CN: β-CN f107−209; α_s1_-I-CN-8P: α_s1_-CN f24−199; α_s1_-I-CN-9P: α_s1_-CN f24−199 9P; A^1^, A^2^, and B: genetic variants of β-CN; 11P, 12P, 9P, 8P, and nP: phosphorylation states of caseins.

Figure 1 shows the electropherograms of the intact CN fractions (α_s1_-CN (8P and 9P), α_s2_-CN (11P, 12P, and nP), β-CN (genetic variants A^1^, A^2^, and B), and para-κ-CN), and their primary hydrolysis products (α_s1_-I-CN (8P and 9P), γ_1_-CN (A^1^ and A^2^), and γ_2_-CN and γ_3_-CN) identified in RO-cheese during ripening.

**Figure 2:**
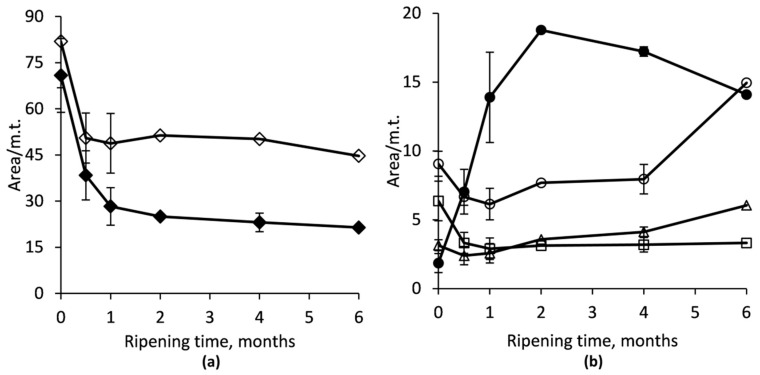
Change of the content (normalized peak area) of main intact CN (**a**) and their primary degradation products (**b**) during RO-cheese ripening. α_s1_-CN (♦): sum of α_s1_-CN-8P and α_s1_-CN-9P; β-CN (◊): sum of β-CN(A^1^), β-CN(A^2^), and β-CN(B); α_s1_-I-CN (●): sum of α_s1_-I-CN-8P and α_s1_-I-CN-9P; γ_1_-CN (○): sum of γ_1_-CN(A^1^) and γ_1_-CN(A^2^); (□): γ_2_-CN; (∆): γ_3_-CN; m.t., migration time.

Intact α_s1_-CN was subjected to more rapid degradation than β-CN: approximately 60% of α_s1_-CN breakdown was observed within the first month and 70% was hydrolyzed by the end of the sixth month of ageing (Figure 1 and Figure 2a). The peaks corresponding to β-CN also showed obvious changes throughout ripening, but still ~55% of the initial β-CN remained intact by the end of the ripening period. The rate of the hydrolysis of the initial CN fraction in RO-cheese was the highest during the first months of ripening (Figure 2).

Due to the lack of published data on RO-cheeses and the large diversity of the manufacturing parameters of traditional cheeses, a direct comparison of the ripening characteristics of our RO-cheese to those of other cheeses was fairly complicated. Nevertheless, it was instantly evident that the course of primary proteolysis in RO-cheese was comparable with those of traditional cheeses. In traditional cheese varieties with similar manufacturing technology, primary proteolysis is characterized by a rapid breakdown of intact caseins to high molecular weight peptides by an activity of both the rennet (chymosin) retained in the curd and the milk native proteinase plasmin. The addition of rennet based on the initial amount of milk used for RO-milk production resulted in an adequate degree of primary degradation of α_s1_-CN in our RO-cheese (Figure 1 and Figure 2a), which is consistent with results on proteolysis in RO-Cheddar cheese [23]. Quite similar results on the breakdown of α_s1_-CN have been reported in a traditional hard cheese Old Saare, with manufacturing and ripening conditions very similar to our RO-cheese (a high curd scalding temperature and both mesophilic and thermophilic LAB as starters), where the same share—~70% of α_s1_-CN—was hydrolyzed during six months of ripening [28]. In 26-week-ripened traditional extra-hard Västerbottenost cheese, manufactured with high scalding temperature and mesophilic starter, the initial peaks of both α_s1_- and β-CN had almost completely disappeared according to the CE profiles [36]. Proteolysis in a typical Gouda-type cheese is determined by an ~70−80% decrease in α_s1_-CN content, mainly by the action of chymosin, already within the first month of ripening [37,38,39], which is consistent with our results for α_s1_-CN degradation; during the production of RO-cheese, the curd scalding temperature was higher (48 °C) compared to Gouda-type cheeses, which could have caused a partial inactivation of chymosin that affected the degradation of α_s1_-CN [40]. Indeed, a rather high percentage of α_s1_-CN breakdown determined in our cheese can be associated with a rather low pH of the cheese environment (in the range of 5.10 to 5.23 during the ripening period) being more favorable for the activity of chymosin as well as also the reversible and incomplete thermal inactivation of chymosin after cooking the cheese curds at 48 °C [40,41].

The substantial contribution of plasmin to the primary proteolysis in cheeses with a high curd cooking temperature is evident [42]. Nevertheless, conversely to α_s1_-CN degradation, significantly less β-CN was hydrolyzed in RO-cheese (45% of intact β-CN hydrolyzed by the end of ripening) compared to the levels reported in Old Saare and even in Gouda-type cheeses (78% and 60−50% of β-CN hydrolyzed by six months of ripening, respectively) [28,37,38,39]. The greater activity of plasmin on β-CN could be expected in RO-cheese, as high cooking temperature inactivates inhibitors of plasminogen activators, leading to the conversion of plasminogen to the active plasmin [43]. Both the higher curd scalding temperature (52 °C) and higher pH during ripening (5.3−5.5) of Old Saare [28], as well as in Västerbottenost cheeses (5.3−5.6) [36], than that of RO-cheese could have exerted an impact on more intensive primary hydrolysis of β-CN in those cheeses [43]. Considerable lower pH values in RO-cheese were evidently decisive for the reduced activity of the alkaline proteinase, plasmin [43], which resulted in impaired hydrolysis of β-CN in RO-cheese.

Large peptides obtained from the primary hydrolysis serve as substrates for subsequent generation of shorter peptides and FAA by complex action of different proteolytic enzymes from LAB [39]. Figure 1 and Figure 2b show that the production rate of chymosin-derived peptide α_s1_-I-CN was the highest during the first two months, while during the next four months of ageing, the degradation rate of that peptide became higher than the production. At the beginning of the ripening, the content of plasmin-derived peptides γ-CNs decreased moderately during the first month, suggesting that they could have been more rapidly degraded further to shorter peptides by the LAB enzymes. The γ-CNs accumulated during further months of aging, showing an increase in the production rate in the period between four and six months (Figure 1 and Figure 2b).

**Figure 3:**
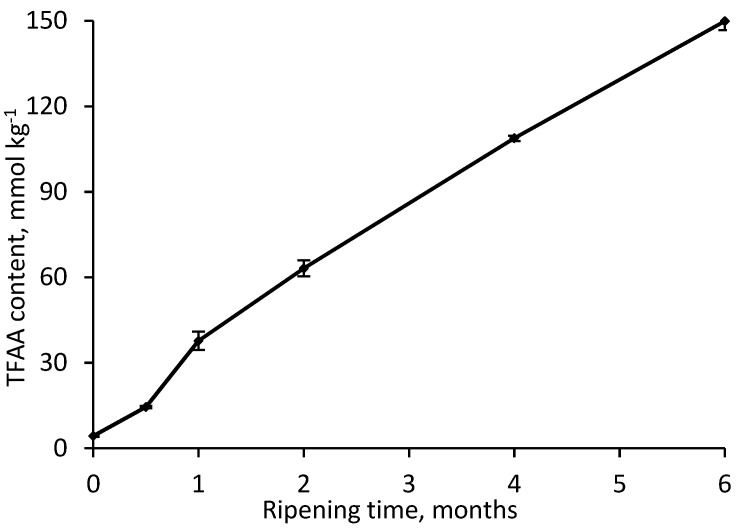
The change in total free amino acids (TFAA) content during RO-cheese ripening.

**Figure 4:**
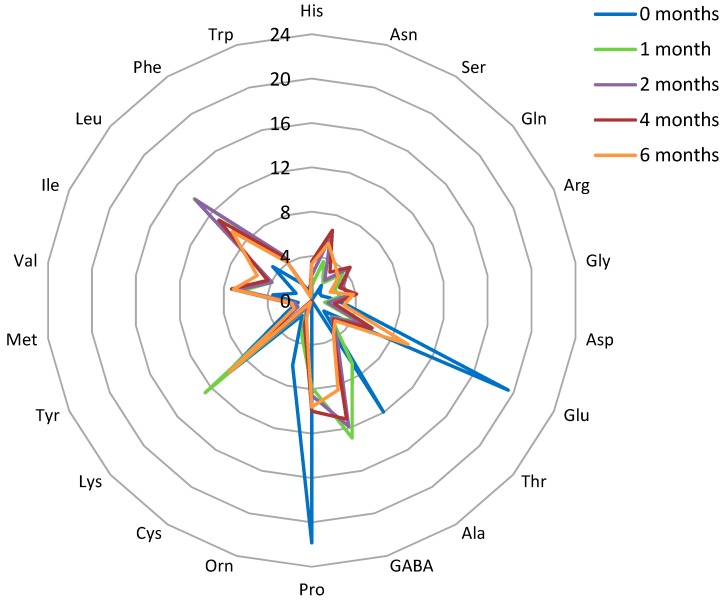
Radar diagram showing the relative content of individual free amino acids (FAA) during six months of RO-cheese ripening (mol%). Results presented are the means of two measurements. Amino acids: GABA, γ-aminobutyric acid; Ala, alanine; Thr, threonine; Glu, glutamate; Asp, aspartate; Gly, glycine; Arg, arginine; Gln, glutamine; Ser, serine; Asn, asparagine; His, histidine; Trp, tryptophan; Phe, phenylalanine; Leu, leucine; Ile, isoleucine; Val, valine; Met, methionine; Tyr, tyrosine; Lys, lysine; Cys, cysteine; Orn, ornithine; and Pro, proline.

The final step of proteolysis is the release of FAA, those enzymatic and chemical conversions to volatile compounds lead to the development of the characteristic cheese flavor [39,44]. The total and relative (mol%) content of FAA released during six months of RO-cheese ripening is shown in Figure 3 and Figure 4, respectively.

The total content of the FAA increased from 4.3 ± 0.3 to 149.9 ± 3.2 mmol kg^−1^ of cheese during maturation. The use of a thermophilic starter in cheese production could have considerably increased the total FAA content [45,46] in RO-cheese. Nevertheless, the level of total FAA in 6-month-old RO-cheese was three-fold lower than that in hard Old Saare cheese (450 mmol kg^−1^ of cheese) [28], it is also made with both mesophilic and thermophilic starters but shows intensive degradation of β-CN, unlike the RO-cheese. Moreover, the total FAA content in RO-cheese was somewhat lower than that in 6-month-old extra-hard Västerbottenost (150−260 mmol kg^−1^) and semihard Herrgård (190 mmol kg^−1^) cheeses manufactured only with mesophilic starters [36,46]. This clearly reveals that the lower total FAA content in RO-cheese can be attributed to the lower degree of hydrolysis of one of the main casein—β-CN—on the first stage of proteolysis mediated by plasmin activity.

The quantitatively dominating amino acids in the ripened 6-month-old RO-cheese cheese were Lys, Pro, Glu, Leu, and γ-aminobutyric acid (GABA) (Figure 4). The first four were found at very similar concentrations (14.2−14.8 mmol kg^−1^ of cheese). It is noteworthy that the same four AA have been shown to be among the quantitatively dominating ones in matured Old Saare cheese [28]. A large amount of Pro (14.4 mmol kg^−1^ in ripened RO-cheese) obviously produced by peptidases of *Lb. helveticus*, may introduce the sweet note in the cheese flavor profile [47]. Amino acid Val, reported to be within the quantitatively dominating amino acids in ripened Old Saare cheese [28], was determined within the dominating ones in the middle of the RO-cheese ripening process (2−4 months), and may contribute to the bitter and sweet flavors of cheese [47]. High levels of Lys and Leu could have contributed to the bitter note [47] in the taste of ripened RO-cheese. GABA was determined in large amounts from the first month of ripening. GABA is not originally present in caseins, but several LABs, including *St. thermophilus* and *Lb. helveticus*, have been shown to exert a GABA-producing ability through the decarboxylation of Glu [47,48].

### 3.2. Volatile Compounds

**Figure 5:**
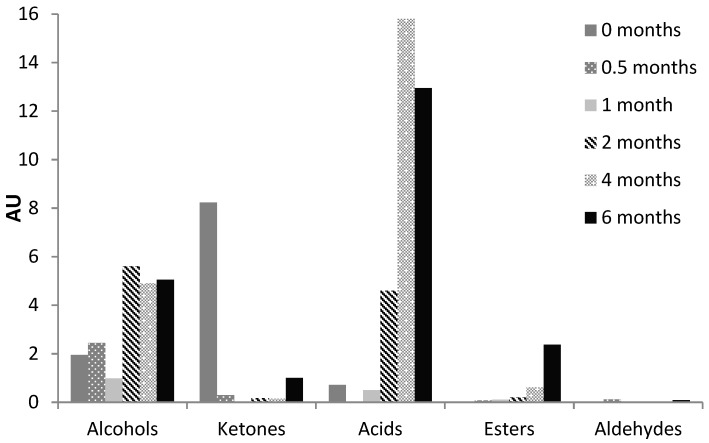
Changes in the content of the main chemical groups of the volatile compounds (AU × 10^4^) identified during RO-cheese ripening. AU: arbitrary units (peak area).

A total of 42 volatile compounds were identified in RO-cheese: nine alcohols, six ketones, seven acids, seven esters, six aldehydes, four aromatic compounds, and three of other groups. Table 2 shows the volatile compounds grouped by classes and the changes in content throughout the ripening process. Volatile acids and alcohols were the main volatiles identified in RO-cheese (1.5−46.3 and 0.0−10.4%TIC, respectively). Esters and ketones comprised the smaller share (0.0−6.7 and 0.0−2.9%TIC, respectively, except for the ketones at the initial point of ripening), while aldehydes and aromatic compounds were among the minor ones (0.0−0.3 and 0.0−0.4%TIC, respectively). The identified volatile compounds showed a dynamic behavior during cheese maturation; however, the total amounts within most detected chemical groups (except for the ketones and aromatic compounds) increased by the end of ripening (Figure 5).

#### 3.2.1. Carboxylic Acids

The total amount of volatile carboxylic acids increased during the ripening of RO-cheese for six months, with a slightly higher content in 4-month-old than in 6-month-old cheese (Figure 5). The relative content of carboxylic acids was the highest in the 4-month-old cheese (46.3%TIC), but also retained high levels toward the end of the ripening period. Acetic acid was the most abundant compound with a very high relative content (1.3−41.0%TIC) during all stages of cheese maturation. The content of acetic acid increased greatly after one month of ripening (Table 2). Acetic and propanoic acids may have a microbial origin and could have been formed as a product of lactose metabolism [44]. Hexanoic acid was present in relatively low levels, exhibiting an ascending tendency solely in the middle of RO-cheese ripening until disappearing after the fourth month of ripening. Butanoic acid was present in comparable amount at the beginning of ripening and showed a substantial increase in amounts up to the fourth month of maturation and disappeared thereafter. Short-chain fatty acids and butanoic and hexanoic acids could be produced as a result of the enzymatic hydrolysis of triglycerides, which is also one of the biochemical pathways essential for cheese flavor development [35]. It is likely that the linear-chain fatty acids could have been transformed into esters [49] since their content was higher at the end of RO-cheese ripening (Figure 5). The main branched-chain fatty acids in RO-cheese—2- and 3-methylbutanoic acid—were present in substantial amounts after four months of ripening (Table 2) and could provide sweaty, sour, fruity, and buttery flavor notes [47,49]. Methylated acids as well as methylated aldehydes and methylated alcohols have a proteolytic origin and, obviously, are produced by the catabolism of branched-chain amino acids (Leu, Ile, and Val) by aminotransferases [47,49]. The prompt decreased in the relative content of Leu in RO-cheese from the fourth month of ripening could be associated with the higher content of 3-methyl-butanoic acid, 3-methyl-1-butanol, and 3-methyl-butanal in ripened 4- and 6-month-old RO-cheeses. In addition, the relative content of Val did not increase much after the second month of ripening, which correlated to the appearance of 2-methylpropanoic acid. Volatiles 2-methylbutanoic acid, 2-methyl-1-butanol, and 2-methyl-butanal originate from Ile, but we could not relate the occurrence of these components in the ripened RO-cheeses to the content of Ile, as it still steadily increased during the RO-cheese maturation. All volatile carboxylic acids identified in RO-cheese (except for propanoic acid) have been previously reported as aroma-active in a wide range of young, medium, and aged traditional Gouda-type cheeses by SPME-GC-Olfactometry [50]. In addition, the same acids (except for acetic and propanoic acid) have been detected in 6-week-old Gouda-type cheeses, while aged cheeses have not shown them at all [51]. Acetic, butanoic, and hexanoic acids, as determined by SPME-GC-Olfactometry, have been found to contribute greatly to the characteristic cheesy sharp and mild to strong savory aroma of typical hard Parmesan and Grana Padano cheeses [52]. Along with the mentioned carboxylic acids, octanoic and decanoic acids were among the abundant aroma compounds in Parmigiano-Reggiano of different ages reported by Bellesia et al. [53].

#### 3.2.2. Alcohols

Alcohols were the second major class of volatile compounds identified in 0-month-old cheese (6.5%TIC) and in cheeses after the second month (up to 10.4%TIC) of ripening (Table 2). The total alcohol content showed an increasing trend throughout cheese maturation up to the second month of ripening with a more rapid change of content after the first month. The total content of alcohols was more or less similar during further RO-cheese ripening (Figure 5). Among the alcohols, 3-methyl-2-butanol was abundant in fresh cheese, whereas in the middle and the later stages of ripening dominated 2,3- and 1,3-butanediol, respectively (Table 2). 2,3-Butanediol could be formed during the citrate or Asp metabolism from 2,3-butanedione (diacetyl) [44], and has been previously reported to be among the important flavor compounds in 0.5- and 4-month-old Gouda cheese [51]. Branched-chain alcohols—2-methyl-1-butanol and 3-methyl-1-butanol—and their corresponding methylated aldehydes—2- and 3-methylbutanal—identified in RO-cheese have been previously detected among the aroma-active compounds in extra-hard Västerbottenost [36], as well as in young and aged Gouda-type cheeses [50,51]. The levels of these compounds were reported to be higher in the matured cheeses, which is consistent with our results obtained for RO-cheese, where these alcohols were detected after four months of ripening. 3-Methyl-2-butanol, 3-methyl-1-butanol, and 2,3- and 1,3-butanediol were detected at trace amounts or among the less abundant alcohols in Parmigiano-Reggiano [53].

#### 3.2.3. Esters

Esters were found in relatively small amounts in the volatile fraction of RO-cheese. This chemical group was presented at a low level from the second week until the second month of ripening and then increased at the later stages of cheese ripening (Figure 5). Butanoic acid ethyl ester and hexanoic acid ethyl ester were dominant among the esters during the entire period of ripening (Table 2). The former was present especially in high amounts in the 4- and 6-month-old cheese and composed 1.6 and 5.7%TIC, respectively. Octanoic acid, decanoic acid, and dodecanoic acid ethyl esters were found only in the 6-month-old cheese. Ethyl esters originate from the enzymatic or chemical esterification of the fatty acids and characterize a cheese by sweet and fruity notes [44]. Butanoic acid ethyl ester and hexanoic acid ethyl ester have been shown to have the highest contribution to the cheese flavor within the esters in young and matured Gouda [50] and hard Parmesan-type and Grana Padano [52,53]. Other ethyl esters identified in RO-cheese have also been quantified, but only in matured Gouda-type cheeses [51], which is in agreement with our results.

#### 3.2.4. Ketones

Ketones were the most abundant class in fresh cheese (35.4%TIC), showing very high amounts compared to the other chemical groups of volatile compounds identified in RO-cheese, mainly because of 2-pentanone (Table 2 and Figure 5). The level of ketones decreased promptly by the second week of ripening. Ketones were found in low levels at the middle stages of maturation. A substantial amount of ketones was observed again at the end of the ripening process in the 6-month-old RO-cheese, mainly because of the increase in content of 2,3-butanedione, which was observed at all stages of ripening. 2,3-butanedione (diacetyl) is one of the products of citrate or Asp metabolism with a sweet buttery aroma [44] and has been demonstrated to be an important aroma-active compound in hard Parmesan cheese [52], considered to be characteristic to Gouda cheeses of different ages [50]. Methyl ketones (2-pentanone, 2-heptanone, and 2-nonanone) can be produced from fatty acids through β-decarboxylation and may be transformed to secondary alcohols [44]. Methyl ketones have been identified as important constituents in blue-cheese aroma [52], although small amounts of those have also been observed in some Gouda [50,51] and Parmigiano-Reggiano of different ages [53]. Moreover, methyl ketones have been found in the fraction of volatile ketones in Västerbottenost cheese with the highest abundance of 2-pentanone [36].

#### 3.2.5. Aldehydes

Aldehydes were present among the volatiles of RO-cheese in very small amounts with a fluctuating behavior during ripening (Table 2). The total content of aldehydes was the highest both in the 0.5- and 6-month-old cheese (0.12 and 0.09%TIC) (Figure 5). Only 2- and 3-methylbutanal were detected in matured 6-month-old cheese, whereas cheeses up to one month of ripening contained the linear-chain aldehydes octanal, nonanal, decanal, and undecanal. Aldehydes are minor volatile components present at low levels in cheese because they are rapidly converted to alcohols or corresponding acids [44]. Aldehydes 2-methyl-butanal and 3-methyl-butanal are the products of the catabolism of branched-chain amino acids Ile and Leu, and have been shown to give the malty, fruity, cocoa, and nutty flavors to cheese [47,49]. The decreasing relative content of Leu in RO-cheese after the fourth month of ripening (Figure 3) can be related to the appearance of low levels of these aldehydes due to their further rapid transformation into the corresponding alcohols 2- and 3-methyl-1-butanol or 2- and 3-methylbutanoic acids (Table 2). The above-mentioned aldehydes have been found among the strong aroma-active compounds at higher concentrations in matured 9- and 10-month-old Gouda than in younger cheeses [50]. In addition, 2-methyl-butanal and 3-methyl-butanal have been detected in Västerbottenost [36] and in some Parmigiano-Reggiano cheeses [53].

#### 3.2.6. Aromatics and Other Compounds

Among the aromatic compounds benzaldehyde, toluene, and acetophenone were found in RO-cheeses up to the fourth month of ripening with a larger share of benzaldehyde in the two first ripening points. Benzaldehyde can be produced from the aromatic amino acid Phe via the α-oxidation of phenyl acetaldehyde and have been shown to give notes of bitter almond to aged Gouda [50,51], Parmigiano-Reggiano [53], and Västerbottenost cheeses [36]. Acetophenone has been found in aged Gouda [50]. Sulfur compounds derive from the amino acid Met and are essential components in many cheeses, giving the boiled cabbage and potatoes, garlic, and egg flavors [47].

### 3.3. Sensory Properties

**Figure 6:**
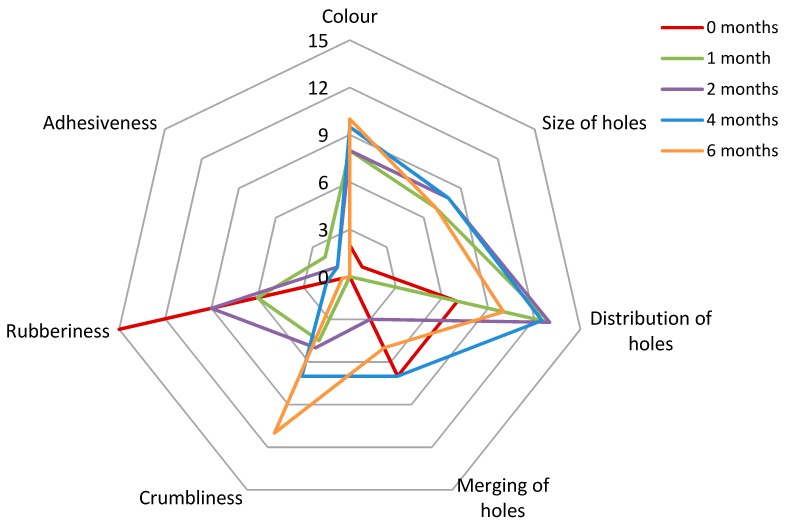
Radar diagram of the appearance and texture attributes of RO-cheese during ripening.

Within sensory perception, an appearance modality revealed the most obvious changes during RO-cheese maturation. The 6-month-old cheese was considerably darker and richer in color than the young RO-cheeses (Figure 6). The size of holes within the RO-cheese matrix grew rapidly during the first month of ripening and then remained relatively the same throughout maturation. The distribution of holes within the cheese matrix became more uniform after the first month of ripening, and some partial merging of the holes was noticed at all ripening stages. The rubberiness decreased, and crumbliness increased dramatically during ripening. In matured 6-month-old RO-cheese, small, white crystals were observed and perceived in the interior of the cheese and on the surface of the holes. The crystals are commonly formed due to the crystallization of amino acids, e.g., Tyr, or calcium lactate, when lactobacilli-containing (including *Lactobacillus helveticus*) starters are used, and have been shown to occur after prolonged maturation of Gouda-type, Cheddar, and Parmesan cheeses [38,54].

**Figure 7:**
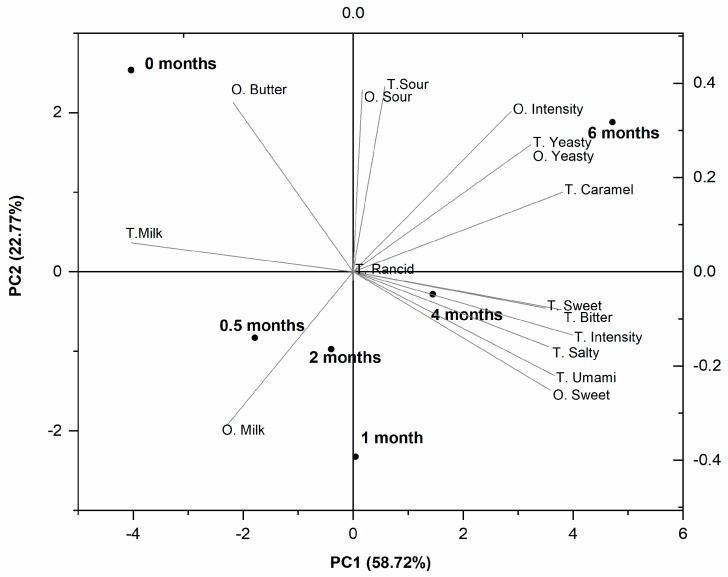
Principal component analysis (PCA) biplot of odor (O) and taste (T) attributes of RO-cheese during ripening. Cheeses are indicated by ripening time. PC: principal component.

Figure 7 shows the principal component analysis (PCA) carried out on the scores of RO-cheese odor and taste evaluation. The first two principal components (PC) explained 81.49% of the variability (PC1: 58.72%; PC2: 22.77%). The odor and taste attributes were related more to the younger, up to 2-month-old cheeses located on the negative axis of PC1, whereas on the positive axis, the attributes received high scores for the matured 4- and 6-month-old RO-cheeses. Young cheeses were characterized by high scores of milky odor and taste and buttery odor, which diminished with maturing time. With maturation, the cheese became more intense in overall intensity (14 out of 15 for odor; 12 for taste), sweetness (8 for odor; 10 for taste), saltiness (seven), and umami taste (seven), and gained low scores for bitterness (three) and caramel taste (two). A slight yeasty odor and flavor (both scored 0.5) were noted in the ripened 6-month-old cheese. These results of the sensory evaluation of odor and taste of ripened 6-month RO-cheese were comparable with those reported for aged 9- and 12-month-old traditional Gouda, where cheeses have been characterized by sweet, salty, and umami tastes and low intensities of caramel and fruity notes [50]. However, an opposite trend in the development of the sweet attribute in cheese maturation, compared to the RO-cheese, has also been observed in Gouda cheese [51].

No defects in descriptive texture and no off-flavor formation were detected during the descriptive sensory analysis of RO-cheese.

## 4. Conclusions

In the present study, the hard high cooked cheese was produced in a pilot plant from 1.9-fold concentrated RO-milk. The RO-cheese produced and evaluated in this study was considered to be of satisfactory quality based on sensory testing by a panel of trained assessors. The evaluation of the primary proteolysis, formation of FAA and volatile compounds proved that the patterns of biological processes essential for cheese maturation which took place in RO-cheese are essentially similar to those in traditional Gouda-type and other hard-type cheeses.

## Figures and Tables

**Table 1:** A complete list of sensory attributes, attribute definitions, reference materials, and their anchor-values on the scale.

Sensory Attributes	Attribute Definition	Reference	
		Commercial 6-Month-Old Old Saare Cheese	Commercial 8-Month-old Gouda Cheese
**Appearance**			
Color	Indicates overall color hue of the sample. The highest score on the scale implies deep orange hue of the sample; the lowest score – off-white hue of the sample	8	15
Hole size	Indicates the average size of holes. The attribute is assessed from the cross-section of a cheese wheel The highest score on the scale implies the presence of large holes in the cross-section; the lowest score—no holes are present in the cross-section of a cheese wheel	0	5
Hole shape	Indicates uniformity and roundness of holes. The attribute is assessed from the cross-section of a cheese wheel. The highest score on the scale implies that the holes (if present) are all round and even; the lowest score—the holes (if present) and misshapen and uneven	0	15
Hole distribution	Indicates the degree of evenness of hole distribution. The attribute is assessed throughout the cross-section of a cheese wheel. The highest score on the scale implies that there is an even distribution of the holes (if present); the lowest score – uneven distribution of the holes (if present)	0	15
Hole merging	Indicates the degree of hole merging (webbing). The attribute is assessed throughout the cross-section of a cheese wheel. The highest score on the scale implies that there is a sever merging of holes; the lowest score—the absence of visible merging	0	0
**Odor** *			
Intensity	Indicates the strength of the overall perceived odor	8	12
Milk	Indicates overall strength of odor characteristic to milk-based products	8	5
Sour	Indicates overall strength of all sour odors	6	3
Sweet	Indicates overall strength of all sweet odors	5	10
Buttery	Indicates the strength of odor sensation characteristic to butter	5	2
Animalic	Indicates the strength of odor sensation characteristic to musky civet and castoreum ^1^	1	0
Sulfur	Indicates the strength of odor sensation characteristic to rotten eggs ^1^	0	0
Animal feed	Indicates the strength of odor sensation characteristic to cattle feed yards ^1^	0	0
Rancid	Indicates the strength of odor sensation characteristic to products of oxidation ^1^	0	0
Yeasty	Indicates the strength of odor sensation characteristic to active yeast ^1^	0	0
Metallic	Indicates the strength of odor sensation characteristic to metal or steel ^1^	0	0
**Flavor** *			
Intensity	Indicates the overall strength of perceived flavor (basic taste + retronasal olfaction)	10	13
Sweet	Indicates the strength of overall sweet sensation (basic taste + retronasal olfaction)	12	8
Caramel	Indicates the strength of retronasal olfaction sensation characteristic to caramel, which is formed as a result of cheese maturation	0	5
Sour	Indicates the strength of overall sour sensation (basic taste + retronasal olfaction) characteristic to acids formed as a result of fermentation	4	6
Bitter	Indicates the strength of bitter taste characteristic to small peptides in cheese (basic taste)	2	3
Salty	Indicates the strength of salty taste characteristic to table salt (basic taste)	5	8
Umami	Indicates the strength of savory taste characteristic to monosodium glutamate (basic taste)	8	6
Milk	Indicates the strength of retronasal olfaction characteristic to milk-based products	8	4
Animalic	Indicates the strength of retronasal olfaction sensation characteristic to musky civet and castoreum ^2^	2	0
Sulfur	Indicates the strength of retronasal olfaction sensation characteristic to rotten eggs ^2^	0	0
Animal feed	Indicates the strength of retronasal olfaction sensation characteristic to cattle feed yards ^2^	0	0
Rancid	Indicates the strength of retronasal olfaction sensation characteristic to products of oxidation ^2^	0	0
Yeasty	Indicates the strength of retronasal olfaction sensation characteristic to active yeast ^2^	0	0
Metallic	Indicates the strength of sensation in the mouth characteristic to metal or steel ^2^	0	0
**Texture**			
Crumbliness	Indicates the number of particles released when breaking the sample in half. The highest score on the scale implies that no particles were released when breaking the sample in half (the sample does not crumble); the lowest score implies a significant release of particles (the sample crumbles)	3	1
Hardness	Indicates the force required to bite through the sample. The highest score on the scale implies that a lot of force is required to make an initial bite through the sample (the samples is hard); the lowest—barely any force is required to bite through the sample (the samples is soft)	5	10
Rubbery	Indicates the rubbery texture characteristic to squeaky cheeses. The highest score on the scale implies that the sample texture is the least similar to that of squeaky cheeses (the samples is not rubbery); the lowest score on the scale implies that the sample texture is similar to that of squeaky cheeses (the samples is rubbery)	3	7
Adhesiveness	Indicates the amount of sample particles that remain on the teeth after chewing the sample for 5 times. For the adhesiveness assessment, a bite of approx. 1 cm × 1 cm piece should be taken. The highest score on the scale implies that the sample leaves behind a significant residue on the teeth after chewing and swallowing (the samples is adhesive); the lowest—no residue is left behind on the teeth after chewing and swallowing (the sample is not adhesive)	2	8

* The highest score on the scale implies very intense sensation; the lowest score – no sensation.^1^ Possible off-odor; ^2^ possible off-flavor.

**Table 2:** Volatile compounds identified in RO-cheese during ripening (AU × 10^4^). Results are the means of three GC-MS measurements. AU, arbitrary units (peak area).

Compound	RT	RI, Exp	RI, Theor ^1^	Odor Description ^2^	Ripening Time, Months					
					0	0.5	1	2	4	6
**Alcohols**										
Isopropyl Alcohol	2.15	510	515	Woody, musty	−	t	−	−	t	0.10
2-Butanol, 3-methyl	7.92	715	700	Musty, vegetable, cheesy	1.34	−	−	−	−	−
3-Buten-1-ol, 3-methyl-	8.33	725	720	Fruity	−	0.04	−	−	−	−
1-Butanol, 2-methyl-	8.34	726	740	Fruity, whiskey	−	−	−	−	−	0.04
1-Butanol, 3-methyl-	8.80	738	750	Fruity, banana, cognac	−	−	−	−	0.09	0.18
2,3-Butanediol	10.34	778	779	Creamy, buttery	0.15	1.72	−	1.73	t	0.86
1,3-Butanediol	10.96	794	789	Odorless	−	−	−	0.32	2.57	2.47
1-Hexanol, 2-ethyl	18.93	1009	1025	Fruity, floral, fatty	0.02	0.03	−	−	−	0.03
1-Undecanol	26.03	1358	1370	Soapy, citrus	−	0.04	−	−	−	−
**Total**					1.51	1.85	0.00	2.05	2.66	3.69
**Aldehydes**										
Butanal, 3-methyl-	5.49	640	652	Fruity, cocoa, nutty	−	−	−	−	−	0.03
Butanal, 2-methyl-	5.78	650	664	Musty, nutty, fermented	−	−	−	−	−	0.06
Octanal	18.49	995	1000	Citrus, orange peel, waxy	−	0.06	−	−	−	−
Nonanal	20.97	1085	1099	Citrus, green, cucumber	0.01	0.03	0.01	−	t	−
Decanal	23.08	1183	1188	Citrus, orange peel, waxy	0.02	−	−	−	−	−
Undecanal	25.07	1295	1310	Soapy, citrus	−	0.02	−	−	−	−
**Total**					0.03	0.12	0.01	0.00	0.00	0.09
**Ketones**										
Acetone	2.12	509	509	Solvent, apple, pear	0.24	0.30	−	−	−	−
2,3-Butanedione	3.68	577	574	Buttery, creamy, milky	0.17	−	−	0.17	−	0.94
2-Pentanone	7.26	697	697	Fruity, banana, fermented	7.79	−	−	−	−	−
2-Butanone, 3-hydroxy	7.42	711	706	Creamy, dairy, butter	−	−	t	−	0.13	0
2-Heptanone	14.00	873	887	Cheesy, fruity, banana	0.04	−	−	−	0.01	0.06
2-Nonanone	20.62	1072	1090	Fruity, dairy, soapy	t	−	−	−	−	−
**Total**					8.23	0.30	0.00	0.17	0.15	1.01
**Acids**										
Acetic acid	5.25	633	640	Pungent	0.71	t	0.47	4.05	14.02	12.62
Propanoic acid	7.11	692	700	Pungent, dairy	−	−	−	−	0.00	−
Propanoic acid, 2-methyl-	9.15	747	758	Acidic, cheesy, dairy	−	−	−	−	0.05	0.04
Butanoic acid	10.14	773	790	Sharp, cheesy	0.01	0.00	003	0.50	1.17	t
Butanoic acid, 3-methyl	12.35	830	848	Cheesy, dairy, fermented, berry	−	−	−	0.00	0.38	0.21
Butanoic acid, 2-methyl	12.74	840	846	Fruity, dairy, cheesy	−	−	−	−	0.05	0.09
Hexanoic acid	17.34	963	990	Fatty, cheesy	−	t	−	0.05	0.11	−
**Total**					0.72	t	0.50	4.60	15.80	12.95
**Esters**										
Ethyl Acetate	4.44	607	610	Ethereal, fruity, grape, cherry	−	t	0.01	−	t	t
Butanoic acid, ethyl ester	10.91	793	798	Fruity, sweet, apple	−	0.07	0.04	t	0.55	2.03
Butanoic acid, butyl ester	18.09	984	994	Fruity, banana, pineapple	−	−	−	0.08	−	0.02
Hexanoic acid, ethyl ester	18.23	988	996	Fruity, banana, pineapple, green	−	−	0.06	0.13	0.06	0.23
Octanoic acid, ethyl ester	23.00	1179	1190	Fruity, pineapple, musty	−	−	−	−	t	0.07
Decanoic acid, ethyl ester	26.27	1373	1381	Fruity, apple	−	−	−	−	t	0.02
Dodecanoic acid, ethyl ester	28.97	1572	1581	Floral, creamy, dairy, soapy	−		−	−	−	0.01
**Total**					−	0.07	0.11	0.20	0.61	2.37
**Aromatics**										
Toluene	9.43	770	774	Sweet, pungent, caramel	t	0.02	0.01	−	0.01	−
Benzaldehyde	16.87	950	955	Almond, cherry	0.06	0.06	0.02	−	0.00	−
Acetophenone	20.17	1055	1062	Almond, cherry, fruity, floral	t	0.02	0.04	0.02	0.02	−
Indole	24.84	1282	1292	Animal, fecal	t	−	−	−	−	−
**Total**					0.06		0.10	0.02	0.03	−
**Others**										
n-Butyl ether	14.05	883	888	Ethereal	−	t	−	0.07	0.01	0.05
Dimethyl sulfone	14.94	897	918	Sulfur	t	0.06	−	−	0.03	0.03
Dimethyl sulfide	2.45	523	530	Sulfur, onion, cabbage	t	0.02	t	−	−	−
**Total**					t	0.08	t	0.07	0.05	0.09

^1^ NIST database (US Department of Commerce, Gaithersburg, MD, USA). ^2^ The Good Scents Company Information System (Oak Creek, WI, USA). t (traces), in nonquantifiable amounts; −, not detected.
